# An Optical Fiber Sensor for Uranium Detection in Water †

**DOI:** 10.3390/bios12080635

**Published:** 2022-08-12

**Authors:** Giancarla Alberti, Maria Pesavento, Letizia De Maria, Nunzio Cennamo, Luigi Zeni, Daniele Merli

**Affiliations:** 1 Department of Chemistry, University of Pavia, Via Taramelli n.12, 27100 Pavia, Italy; 2 Ricerca sul Sistema Energetico-RSE S.p.A, Via R. Rubattino n.54, 20134 Milan, Italy; 3 Department of Engineering, University of Campania Luigi Vanvitelli, Via Roma n.29, 81031 Aversa, Italy

**Keywords:** optical sensor, surface plasmon resonance (SPR), plastic optical fiber (POF), uranyl analysis, environmental waters, out-of-the-lab analysis

## Abstract

An optical sensor for uranyl has been prepared based on a gold-plated D-shaped plastic optical fiber (POF) combined with a receptor consisting of a bifunctional synthetic molecule, 11-mercaptoundecylphosphonic acid (MUPA), with a phosphonic group for complexing the considered ion, and a sulfide moiety through which the molecule is fixed at the gold resonant surface as a molecular layer in an easy and reproducible way. The sensor is characterized by evaluating the response in function of the uranyl concentration in aqueous solutions of different compositions and real-life samples, such as tap water and seawater. The mechanism of the uranyl/MUPA interaction was investigated. Two different kinds of interactions of uranyl with the MUPA layer on gold from water are observed: a strong one and a weak one. In the presence of competing metal ions as Ca^2+^ and Mg^2+^, only the strong interaction takes place, with a high affinity constant (around 10^7^ M^−1^), while a somewhat lower constant (i.e., around 10^6^ M^−1^) is obtained in the presence of Mg^2+^ which forms stronger complexes with MUPA than Ca^2+^. Due to the high affinity and the good selectivity of the recognition element MUPA, a detection limit of a few μg L^−1^ is reached directly in natural water samples without any time-consuming sample pretreatment, making it possible for rapid, in situ controls of uranyl by the proposed sensor.

## 1. Introduction

Optical sensors exploiting the surface plasmon resonance phenomenon on multimode optical fibers for the specific detection of chemical species of biological interest have been widely demonstrated in the last few decades, as reported in many review papers [1,2,3,4]. In particular, our research group proposed a surface plasmon resonance (SPR) optical platform based on a multimode plastic optical fiber (POF) which is relatively fast and easy to prepare and requires a low cost and low dimensional apparatus. The plastic optical fiber has a characteristic D-shaped profile simply obtained by erasing a multimode POF as it was described over ten years ago [5] with a multilayer plasmonic resonant surface. The sensor is obtained by combining the surface at which the plasmonic resonance occurs with a specific molecular recognizing element (MRE) fixed as a layer in tight contact with the surface.

With the aim to reach low detection limits and high selectivity, different MREs were applied in combination with this platform, both biomolecules, for example, proteins or aptamers [6,7,8] and synthetic receptors, as molecularly imprinted polymers (MIPs) [9]. The possibility of using this particular kind of POF platform for the detection and quantification of several biomolecules [6,7,8], as well as many small molecules of interest in different fields was demonstrated: for example, in defense [9], environment [10] and food control [11]. In a few cases, the same platform (SPR-POF) has been proposed for the detection of metal ions, such as iron(III) [12] and copper(II) [13]. In these cases, the MREs were synthetical ligands of the considered metal ion, as deferoxamine for iron(III) and penicillamine for copper (II), for which a detection limit of about 10^−6^ M, or fraction of mg L^−1^, is demonstrated. This kind of MRE can be regarded as *biomimetic* because of the strength of the specific interaction with the considered metal ion and the relatively good selectivity.

Uranium is a radioactive element with an estimated mean concentration of 2.7 mg kg^−1^ in the Earth’s crust and 3.3 µg L^−1^ in seawater [14,15]. It is radio and chemically toxic and is present in the environment in different chemical species; however, the main form is uranyl, UO_2_^2+^, a double charged ion in which uranium is at +6 oxidation state. It has some important industrial applications, particularly in the defense field, since the by-product of the uranium enrichment process, the depleted uranium (DU, i.e., the isotope ^238^U), was applied as armor-piercing ammunition in several international military conflicts because of its high density and hardness. The use of such ammunition has led to the release of DU into the environment worldwide, raising the diffusion of toxic ^238^U to the environment. Thus, the possible increased presence of uranium in environmental waters has recently attracted considerable public interest.

Classical methods for metal detection at low concentration levels have been successfully applied, and sensing methods have been considered too [16,17,18,19], particularly to perform out-of-lab analysis, reducing time and costs. Optical sensing methods have attracted considerable interest in the last few decades, obtaining brilliant results in terms of low detection limits, as seen, for example, using a uranyl-specific DNAzyme as an MRE [18,19]. 

In this work, the D-shaped SPR optical platform (SPR-POF) previously developed by our group is used to obtain a specific sensor for uranyl detection in water, to demonstrate the sensing approach’s capability for the determination in real aqueous samples in the µg L^−1^ range. The MRE was 11-mercaptoundecanphosphonic acid (MUPA), a very efficient receptor for uranyl due to the presence of the phosphonic moiety, as previously demonstrated in an electrochemical sensor [17]. At the same time, it can be easily linked to the gold surface of the optical platform by self-assembling via the –SH group and, being a simple synthetic molecule, is less costly and more stable in out-of-lab applications than biological molecules. Regarding the possibility of realizing the proposed sensing approach, preliminary results have been presented in [20].

The sensing device proposed here is interesting because, in principle, it can be applied directly in the field, giving an analytical response in a fast and not too expensive way. Indeed, the SPR transduction method is marker-free, so that it can be applied to different metal ions, even not electroactive as in the case of electrochemical transduction, provided that a proper MRE is fixed at the SPR interface.

## 2. Materials and Methods

### 2.1. Materials and Reagents

Reagents of the purest grade available were purchased from Sigma-Aldrich and used as received. All the solutions were prepared with bi-distilled water (DI) water. Uranyl standard (Aldrich, standard for ICP-OES, 1000 mg L^−1^) was used to prepare daily diluted standard solutions. 11-mercaptoundecylphosphonic acid (MUPA) was obtained from Sigma-Aldrich. To avoid contamination, all glassware was carefully cleaned with concentrated nitric acid and then rinsed with DI water before use.

### 2.2. SPR-POF Platform Preparation

A polymeric optical fiber with a core of poly-methylmethacrylate (PMMA) with 980 µm diameter and a cladding of fluorinated polymer of 10 µm (1 mm total diameter), embedded in a resin support, as previously described [5,10,13], was used to realize the optical sensor platform. A D-shaped region was produced on the POF by removing the cladding and part of the core, along the half circumference, by polishing with two kinds of polishing papers. Then, a Microposit S1813 photoresist was spun (6000 rpm for 60 s) on the exposed POF core, using a spin coater machine to obtain a layer about 1.5 µm thick between the POF core and the metal. This layer has a refractive index higher than that of the POF core and can considerably improve the performance [5]. Finally, a thin gold film was sputtered by a Bal-Tec SCD 500 machine. The sputtering process was repeated three times by applying a current of 60 mA, at 0.05 mbar, for 35 s to obtain a 60 nm thick layer (20 nm of gold for each step). The optical platforms prepared as described are indicated as SPR-POF.

### 2.3. MUPA Deposition

**Figure 1:**
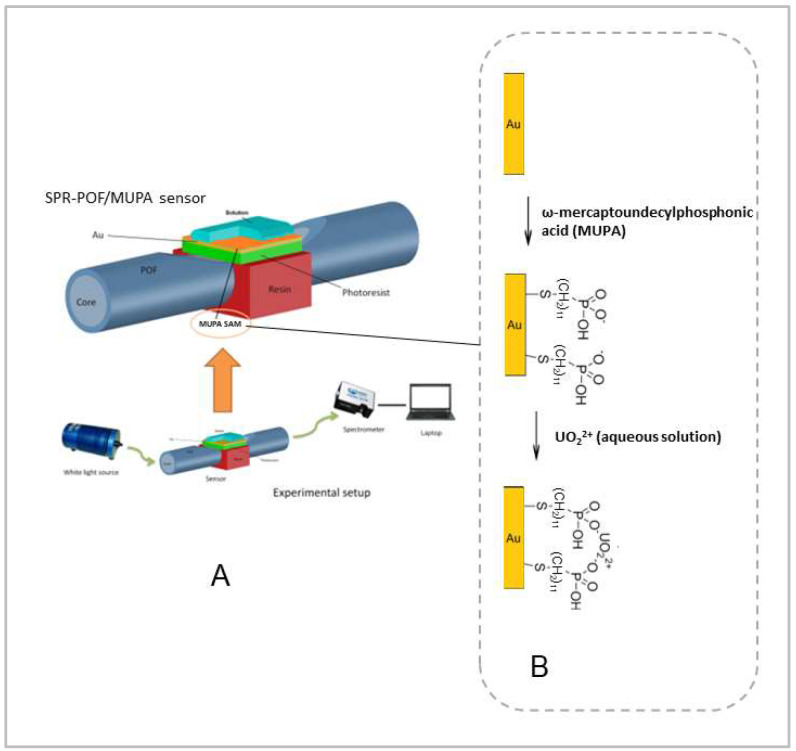
Scheme of the experimental setup (**A**) and the MUPA immobilization on the gold layer of the sensor together with the binding step (**B**).

The selected ligand for uranyl, i.e., MUPA, was immobilized as a self-assembling monolayer over gold, taking advantage of the presence of the thiol group, according to the previously described procedure [17], used to realize a sensor with electrochemical transduction. Briefly, the gold layer was contacted overnight with a solution containing 2.5 mM MUPA in 20% methanol/80% water. The modified sensor was then abundantly rinsed with ethanol and Milli-Q water before use. The sensors derivatized with MUPA are indicated in the text as SPR-POF/MUPA. An outline of the MUPA layer deposited on the gold surface of the sensor together with the binding step is reported in Figure 1.

### 2.4. Experimental Setup

The white light source was the Halogen lamp HL-2000-LL (manufactured by Ocean Optics, Dunedin, FL, USA), and the spectrometer was a FLAME-S-VIS-NIR-ES, manufactured by Ocean Optics, Dunedin, FL, USA. The white light source presents an emission range from 360 nm to 1700 nm, whereas the spectrometer has a detection range from 350 nm to 1023 nm. The transmission spectra and data values were displayed online on the computer screen and saved by Spectra Suite software (Ocean Optics, Dunedin, FL, USA). The spectra were normalized by the Matlab software (MathWorks, Natick, MA, USA) using as reference for normalization the spectrum acquired with air as a surrounding medium over the bare gold surface or the MUPA derivatized gold surface. Figure 1 shows a scheme of the experimental setup and an outline of the SPR-POF probe combined with the MUPA layer.

### 2.5. Measurements

The measurements were performed in a drop since this procedure requires a more compact and much less expensive apparatus than the flow method. About 50 µL of the aqueous sample solution were dropped over the sensing region and incubated at room temperature for 10 min. The spectrum was acquired, normalized and plotted to evaluate the minimum transmission wavelength (the resonance wavelength).

The quantity of analytical interest is the resonance wavelength variations with respect to the resonance wavelength of the blank solution (Δ*λ*_res_). The function Δ*λ*_res_ vs. uranyl concentration (*c*) were fitted to Equation (1), i.e., the Hill equation [21], using the software OriginPro (Origin Lab. Corp., Northampton, MA, USA) and assuming that the third parameter of the Hill equation in OriginPro, *n*, is equal to 1.

The Hill model corresponds to the Langmuir adsorption isotherm so that the response of the sensor to the analyte concentration is as follows:

(1) $$\Delta \lambda_{\mathrm{res}}=\lambda_{c}-\lambda_{0}=\frac{\Delta \lambda_{\max}\cdot c}{K_{d}+c}$$

where *c* is the analyte concentration, Δ*λ*_max_ is the value of the maximum resonance wavelength variation at increasing concentration of uranyl, i.e., the value at saturation. The symbol *λ*_c_ indicates the resonance wavelength at *c* concentration and *λ*_0_ at 0 concentration. *K*_d_ corresponds to the dissociation constant, i.e., the reciprocal of the affinity constant *K*_aff_, of the molecular recognition elements in the Langmuir adsorption model. Equation (1) holds for one kind of interaction sites but can be extended to the case of more than one affinity site. For example, it has been successfully used in the case of D-Shaped POF sensors with MIP as a molecular recognition element [9]. Equation (1) can be used as the calibration curve, making it possible to extend the calibration range beyond the linearity range.

All measurements were made at 25 °C. Throughout the paper, the standard deviation between parentheses refers to the uncertainty of the last digit.

## 3. Results

The optical sensitivity of the D-shaped SPR platforms considered here was evaluated as previously suggested [5], obtaining values similar to those found for the D-shaped POF optical platforms examined elsewhere. The optical sensitivity increases at an increasing refractive index (RI), but the relation *λ*_ris_ vs. RI can be considered linear in small *λ* ranges. At the refractive index of water (about 1.33), the sensitivity is 1700 RIU cm^−1^, suitable for sensing applications.

### 3.1. SPR Characterization of the Sensor

A preliminary analysis was carried out to monitor the formation of the receptor self-assembled monolayer by exploiting two different normalization approaches of the acquired SPR spectra.

**Figure 2:**
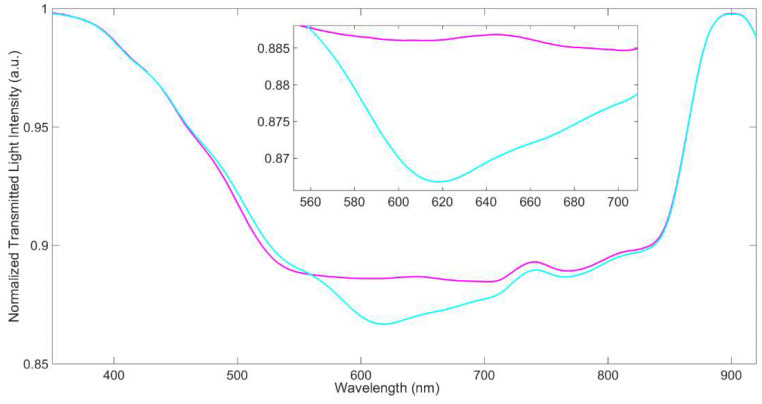
Transmission spectra of SPR-POF/MUPA in water (light blue line), and in air (purple line), normalized on SPR-POF in air.

The first reference spectrum considered is that of SPR-POF with a bare gold surface in air, acquired before the functionalization process, and can be used to monitor if any change of the optical guiding, specifically of the waveguide effective index, takes place after the functionalization process. To obtain this information the SPR spectra acquired with water and air as surrounding media on the MUPA SAM are normalized on the spectrum obtained by the same SPR-POF probe before the functionalization process (bare gold surface) in air. This normalization process is based on the ratio of transmitted spectra (gold surface covered by MUPA SAM in water or in the air) to the transmitted spectrum in the air of a different optical waveguide (gold surface without MUPA SAM) with a diverse effective index. Such spectra are reported in Figure 2. So, the normalized transmitted spectra show that the optical guiding effect is reduced in the range from about 500 nm to 800 nm when the refractive index of the dielectric medium in contact with the gold surface increases. Moreover, Figure 2 shows that the normalized SPR spectrum in the air of the chip covered by MUPA SAM is “flat” in the range from 520 nm to 710 nm with a normalized transmitted light intensity value lower than unity, indicating that just the optical guiding effect is reduced. On the other hand, when water is present on the MUPA SAM surface, a dip at about 618 nm is present, indicating that an SPR phenomenon is also triggered, together with the reduction in the optical guiding effect.

**Figure 3:**
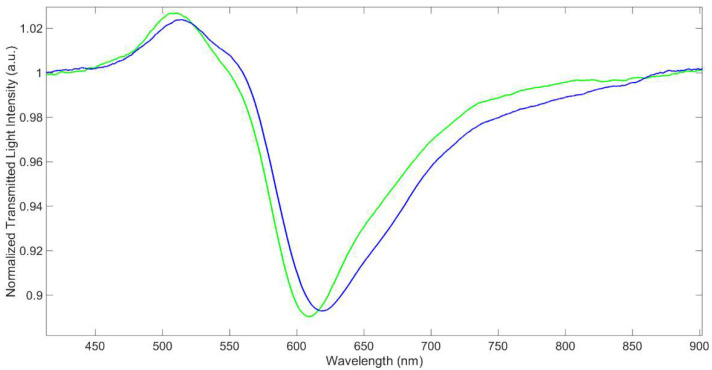
Transmission spectra of SPR-POF (green line) and SPR-POF/MUPA (blue line) in water normalized to the spectrum of the corresponding platform in air.

From these results, we can conclude that the SPR-POF/MUPA sensor in air does not present any plasmonic resonance, even if the effective index of the optical waveguide changes in comparison with that of SPR-POF. This means that the spectrum in air can be used as a suitable reference to normalize the SPR spectra in fluids, such as water, with a higher refractive index. Consequently, the SPR spectrum of the SPR-POF/MUPA in water has been normalized on that of the SPR-POF/MUPA in air, in a similar way to the normalization approach used for the bare surface (spectrum in water normalized on that acquired in the air), as reported in Figure 3.

Moreover, the resonance wavelength at SPR-POF/MUPA (about 618 nm) is red-shifted with respect to that at the bare gold surface, which is at 604.3 nm, as seen in Figure 3. This indicates that the MUPA layer is actually present over the gold surface. A similar effect was previously noticed in D-shaped POF sensors based on receptors in the form of a molecular layer [7,12,13] or a thin polymeric film [9]. 

The depth of the resonance peak in water is about 11% in both the optical platforms and the FWHM (Full Width Half Minimum) is, respectively, 80 and 90 nm, i.e., somewhat larger for the derivatized sensor; however, the minimum is still clearly detectable.

### 3.2. Reproducibility

**Figure 4:**
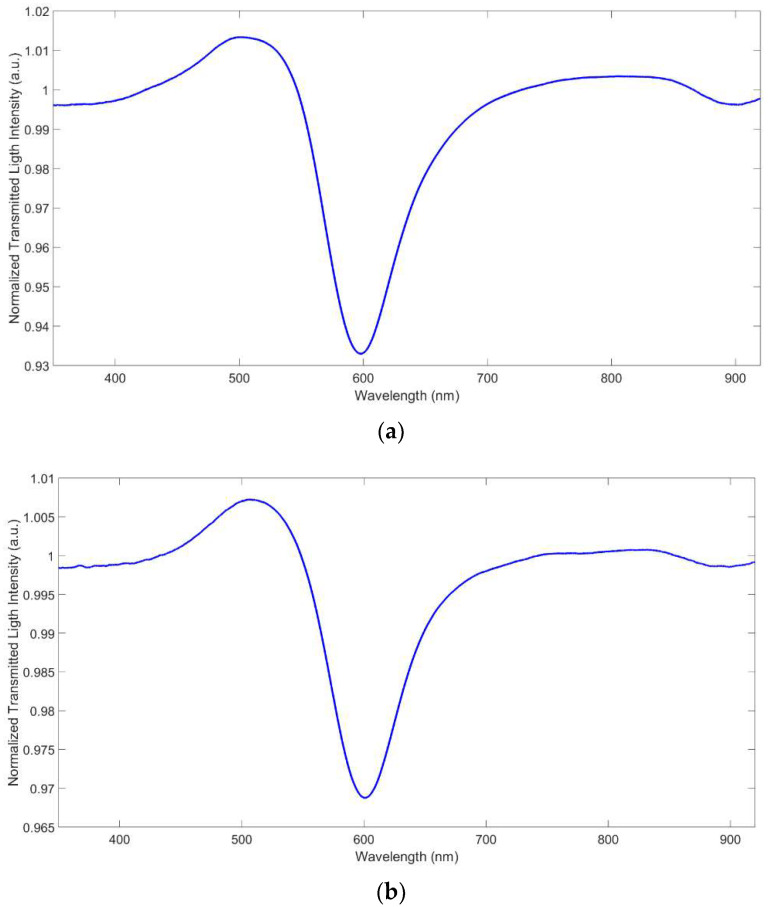
Transmission spectra in water of two different platforms, SPR-POF S3 (**a**) and S4 (**b**), normalized to the spectra of the same sensor in air.

The D-shaped POF platforms utilized here are prepared in a way that a large irreproducibility can be expected, and that can be evaluated by comparing the resonance wavelength in water for different sensors, as reported in Figure 4. The depth of the peak is considerably irreproducible too. Moreover, the deposition of the MRE could also produce a further irreproducibility. Four different MUPA-derivatized sensors, prepared with the same procedure, gave similar spectra in water with a resonance wavelength (*λ*_res_) in water of 604.6(4.9) nm (mean value). 

The slight variation is due to the not-perfect reproducibility of the handmade platforms used here. *λ*_res_ in water of the corresponding MUPA-modified platforms is 611.5(3.6) nm. The fact that the standard deviation of the bare and MUPA-modified sensors is similar indicates that the receptor layer is formed in a reproducible way that does not contribute to the global irreproducibility of the system. Another source of irreproducibility is the experimental setup, due for example to the connection between the light source and the spectrometer. 

After deposition of MUPA on the gold surface, *λ*_res_ in water is red-shifted with Δ*λ*_res_ = 6.8(2.8) nm. The reproducibility of the red shift is slightly better than that of the resonance wavelength since the irreproducibility due to platform variations is cancelled, while that due to the setup still persists.

### 3.3. Sensitivity to the Refractive Index of the Aqueous Sample

In the case of very thin layers, as the monolayers or quasi-monolayers here considered [17], the sample’s refractive index overlaying the sensing surface can influence the sensor response. Pure water has an RI suitable for measurement with the D-shaped POF platforms proposed here. However, aqueous solutions can have RIs noticeably higher than that of pure water, depending on the solution composition.

In some cases, for example in human serum, the RI is so high that direct measurements are even impossible [22]. In this work, a solution NaNO_3_ 0.1 M was considered for characterization, with RI = 1.3337, while the RI of water at the same conditions was 1.3327 [23]. A red shift of 7.7 nm in NaNO_3_ 0.1 M with respect to pure water was recorded at a bare sensor SPR-POF while only 2.3 at SPR-POF/MUPA. This shows that the bare sensor has higher sensitivity to the RI of the overlaying dielectric compared to that with MUPA, as also observed in the case of a polymeric receptor layer [9], which is expected because of the existence of the receptor layer over gold.

Natural freshwaters are relatively simple matrices with low ionic strength and have a variable ionic composition, so a relatively concentrated medium (NaNO_3_ 0.1 M) was selected for aqueous measurements to buffer the ionic composition of the samples under investigation.

Besides influencing the refractive index of the sample, the neutral and ionic dissolved substances can influence the refractive index of the receptor layer by interacting with it. In particular, cations can be adsorbed by MUPA fixed at the gold surface by an ion exchange reaction with the protons linked to the phosphonic groups, modifying the receptor layer’s chemical composition and structure. A high concentration of a known salt, such as NaNO_3_, can fix the refractive index of the sample and at the same time determines the chemical structure of the receptor layer, which in this way is substantially independent of the original composition of the sample.

### 3.4. Characterization of the Sensor Response to Uranyl Ion

**Figure 5:**
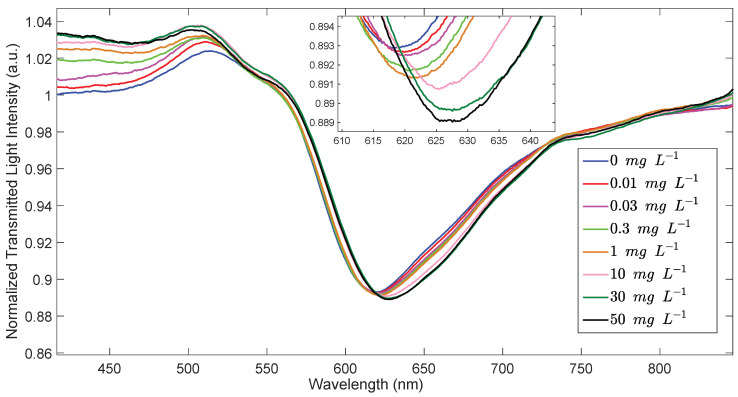
Transmission spectra of SPR−POF/MUPA in NaNO_3_ 0.1 M aqueous solution of uranyl at different concentrations.

The resonance wavelength of SPR-POF/MUPA is red-shifted at increasing concentrations of uranyl as reported in Figure 5.

**Figure 6:**
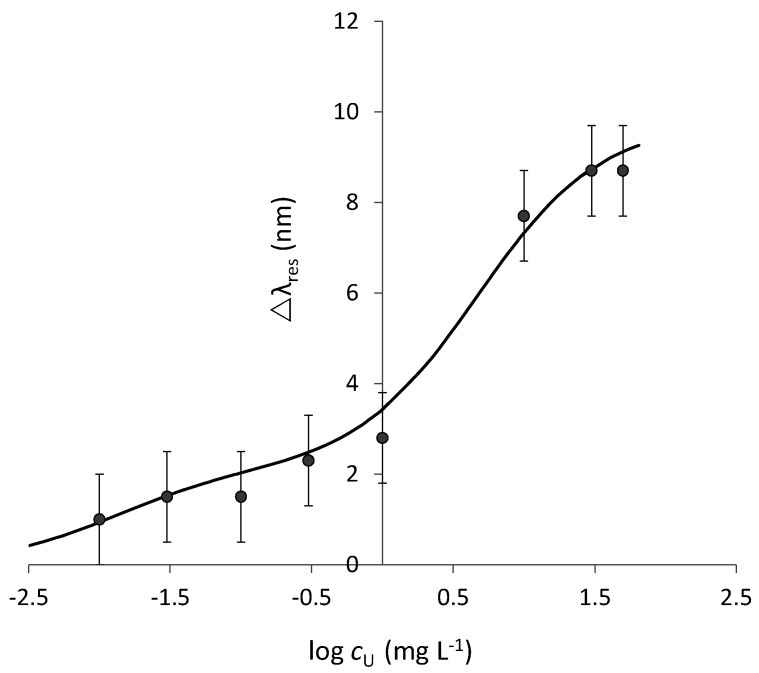
Standardization curve of uranyl in 0.1 M NaNO_3_ aqueous solution at SPR−POF/MUPA.

The variation of the resonance wavelength (Δ*λ*_res_) in function of the uranyl concentration in aqueous solution (*c*), evaluated as the shift from the resonance wavelength of the blank sample (not containing uranyl ion), is reported in Figure 6 in a linear/log scale.

The experimental data reported in Figure 6 shows two plateau values, indicating that two kinds of adsorbing sites are present in the considered adsorbing layer despite a single receptor (MUPA) being linked to the SPR active surface. The stronger sites (site S) interact with uranyl at a lower concentration up to about 0.300 mg L^−^^1^. Then, when these sites are saturated, the weaker ones (site W) are occupied too by uranyl. To consider the possible presence of two sites with a different affinity, Equation (2) is used to fit the data. It is an extension of the Hill equation (Equation (1)) when two different combination sites are present. The dissociation constant of the two sites, strong and weak, are indicated by the suffix S and W, respectively.

(2) $$\Delta \lambda_{\mathrm{res}}=\frac{\Delta \lambda_{\max,S}\cdot c}{K_{S}+c}+\frac{\Delta \lambda_{\max,W}\cdot c}{K_{W}+c}$$

The parameters for the two sites, obtained by Solver of Microsoft Excel, are reported in Table 1.

The lower detection limit (LOD) is acceptable considering the possible applications in drinking water, where a limit of 30 µg L^−^^1^ for uranium is suggested [14]. Instead, this limit can be considered too high for the quantitative determination of uranium in not contaminated environmental waters, where concentrations lower than 1–3 µg L^−^^1^ are often present [19]. 

The LOD (expressed as molarity) obtained with the sensor presented here is two orders of magnitude lower than that found for a similar sensor for Cu^2+^ [13], which is evidently due to the much higher affinity of uranyl for the receptor MUPA (*K*_aff_ = 1.8·10^7^ M^−^^1^) than of Cu(II) for its ligand (penicillamine; *K*_aff_ = 4.7·10^4^ M^−^^1^). 

As far as the lower concentrations are concerned, the higher quantification limit is 11 µg L^−^^1^ (calculated as (Δ*λ*_max_-SE)/sens). Nevertheless, a response is obtained at higher concentrations too, due to the sites with lower affinity for uranyl in the MUPA layer fixed at the sensor surface. After the saturation of the sites at higher affinity, those at lower affinity are occupied too, but only at higher uranyl concentration. The weaker sites have a detection limit of 0.44 mg L^−^^1^. Thus, the proposed sensor cannot quantify the uranyl concentrations between 11 and 440 µg L^−^^1^.

### 3.5. Interferences by Ionic Components of the Sample

**Figure 7:**
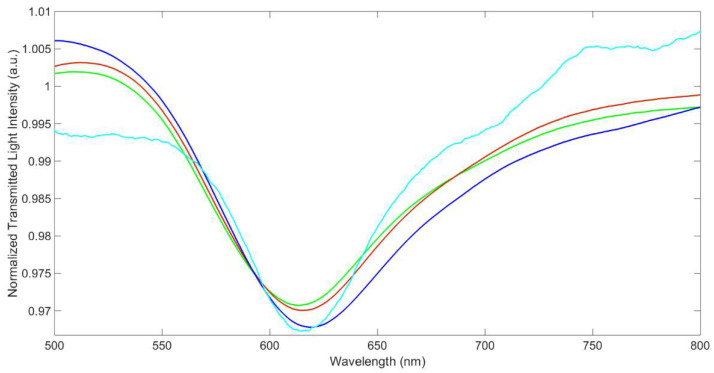
Transmission spectra of SPR−POF/MUPA A2U in aqueous samples. green line: water; red line: NaNO_3_ 0.1 M water; blue line: 625 mg L^−1^ Ca^2+^ (15.6 mM) in NaNO_3_ 0.1 M solution; light-blue line: 225 mg L^−1^ Mg^2+^ (9.1 mM) in NaNO_3_ 0.1 M.

Some interference is expected in the sensor response, in particular from other metal ions, which can be competitively adsorbed by the MUPA layer, producing a variation of the refractive index of the dielectric in contact with the gold film in the same way as the adsorption of uranyl. Cations can be adsorbed on the MRE, by ion exchange or by complexation by the phosphonic groups of MUPA, as in the case of UO_2_^2+^, inducing a change in the chemical composition and the structure of the receptor layer and so of the resonance wavelength. For example, Na^+^ can only be exchanged with H^+^, while alkaline earth metal ions, such as Mg^2+^ and Ca^2+^, can be complexed by the phosphonic groups of MUPA, having a high affinity for phosphate/phosphonate moiety [24]. Since these cations are widely present in environmental waters, they are examined here in detail. For example, Figure 7 shows the transmission spectra of the sensor in a solution containing Ca^2+^ and Mg^2+^ (at concentrations near those in drinking waters) compared with those in water and NaNO_3_ 0.1M.

The resonance wavelength in water at SPR-POF/MUPA is at 613 nm, while that in 0.1 M NaNO_3_ is shifted to the higher wavelengths by 2 nm. The observed shift can be ascribed to the variation of the RI of the solution and to the variation of the RI of the MRE layer due to the ion exchange of H^+^ with Na^+^, since the atomic mass of Na^+^ (22.9 g/mol) is higher than that of H^+^ (1 g/mol).

A further slight increase of the resonance wavelength is observed for the ion exchange of H^+^/Na^+^ with Ca^2+^ (atomic mass 40.0 g/mol) and Mg^2+^ (atomic mass 24.3 g/mol), even if these metal ions are at a much lower concentration than Na^+^, as was observed above in the case of uranyl ion (molecular mass 270.03 g/mol).

**Figure 8:**
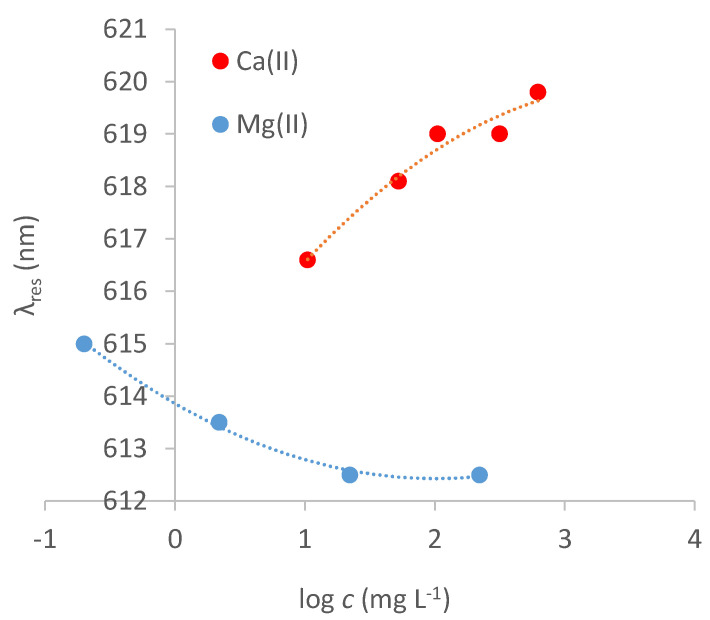
Dose−response curve of Ca^2+^ (red points) and Mg^2+^ (blue points) at SPR−POF/MUPA in NaNO_3_ 0.1 M water solution.

The dose–response curves of Ca^2+^ and Mg^2+^ on the MUPA sensor are reported in Figure 8. Δ*λ*_res_ strongly depends on the concentration of the metal ions in the solution phase, reaching a steady value at a concentration higher than 300 mg L^−^^1^ in the case of Ca^2+^ and 10 mg L^−^^1^ in the case of Mg^2+^.

An interesting difference in the response of the two metal ions is that when the concentration of Mg^2+^ increases, the resonance wavelength decreases while it increases in the case of Ca^2+^, similar to uranyl. This behavior is probably due to calcium and uranyl’s much higher mass than that of magnesium. Actually, an exchange of two Na^+^ (atomic weight 22.9 g/mol) for one Mg^2+^ produces a variation of −21: while for calcium, +5.8; and for uranium, +51. The observed behavior can also be due to a conformational change of the receptor layer in the presence of the metal ions, such as a shrinking in the presence of Mg^2+^, which is a small double charge cation, and a swelling in the case of larger ions, as Ca^2+^ and uranyl, or mono-charged ions, as Na^+^.

As can be observed in Figure 8, in the case of Mg^2+^ and Ca^2+^, only one kind of complex appears to be formed at the MRE so that the sensor’s response to these cations can be modelled according to Equation (1). The obtained parameters are reported in Table 2.

The *K*_aff_ value for Mg^2+^ is two orders of magnitude higher than that of Ca^2+^. The difference is much higher than that of the affinity constants in aqueous media of ligands similar to MUPA [24], indicating that different effects influence the formation of complexes at the monolayer-solution interface compared to those in the solution phase. Both *K*_aff_ values are lower than that of uranyl for the strong sites. Compared with the weak sites’ affinity for uranyl, the *K*_aff_ for Ca^2+^ is lower while it is higher in the case of Mg^2+^.

**Figure 9:**
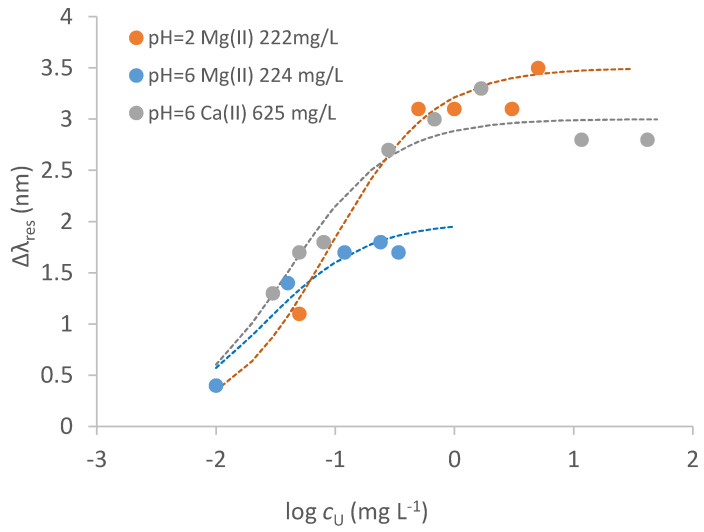
Calibration curves of uranyl in NaNO_3_ 0.1 M solution with Mg^2+^ (222 mg L^−^^1^, pH = 2 orange points; 224 mg L^−^^1^, pH = 6 blue points) and Ca^2+^ (625 mg L^−^^1^, pH = 6 grey points) on SPR−POF/MUPA sensor. Dotted lines are the calculated curves applying Equation (1) (parameters in Table 3).

The presence of ions such as Na^+^, Mg^2+^ and Ca^2+^ is relevant in determining the resonance wavelength of the blank solution in natural waters, even at low concentrations. Thus, the reference solution for calculating the signal, Δ*λ*_res_, must have the same ionic composition of the sample solution. Some standardization curves of uranyl in solution with different concentrations of Mg^2+^ and Ca^2+^ and at different acidities are reported in Figure 9. 

Table 3 summarizes the parameters of the dose–response curve of uranyl in the presence of Ca^2+^ or Mg^2+^, evaluated by Equation (1), considering that only one kind of complex seems to be formed at the considered conditions. Since the affinity constants of Ca^2+^ and Mg^2+^ are much lower than that of uranyl (see Table 2), the presence of such ions is expected to have a marginal influence on the uptake of uranyl when it interacts with the strong sites, and so on the signal at the concentrations generally present in low salinity waters. Some competition could occur but at higher concentrations of the competing metal ions. 

The affinity constant in the presence of Mg^2+^ and at acidic pH is one magnitude order lower than in the absence of it, indicating that Mg^2+^ competes with uranyl more than Ca^2+^. Nevertheless, the LOD of uranyl in the presence of Mg^2+^ is only slightly higher. The effect of Ca^2+^, even if present at a slightly higher concentration, is much lower, with *K*_aff_ being only three times lower than that in the absence of Ca^2+^. The LOD in the presence of interfering ions is acceptable for many applications, considering the concentrations at which uranyl can be present both in natural and contaminated waters [14].

Calcium, which is known to form strong complexes with phosphonates [25], and magnesium, did not interfere even at Ca^2+^ or Mg^2+^ to uranyl weight ratio 1000 to 1 [17]. However, another possible interference could be given by the combination of calcium and carbonate with uranyl leading to stable soluble compounds, such as CaUO_2_(CO_3_)_3_^2−^ and Ca_2_UO_2_(CO_3_)_3_ [26]; this could cause some interference in environmental waters in which all these species co-exist.

An important parameter determining the sensor’s response is expected to be the sample’s acidity. Indeed, a variation of the acidity may produce a change in the complexing environment of the MUPA monolayer, since MUPA is partially deprotonated in the pH range 3–6 and fully deprotonated at higher pH. Moreover, the solution acidity can influence the formation of metal complexes, including hydroxyl derivatives in aqueous solution.

For example, acidifying the solution containing 220 mg L^−^^1^ Mg^2+^ at pH = 2, the resonance occurs at *λ*_res_ = 609.0 nm instead of 612.5 nm when the solution is at natural pH, i.e., around 7. In this case too there is a blue shift, possibly due to the exchange of Na^+^ and/or Mg^2+^ with H^+^ as reported above.

Similarly, it is expected that both acidity and the presence of metal ions affect the strength of the interaction of uranyl with the sensing surface, and so the resonance wavelength. In particular, the formation of uranyl hydroxo complexes in water solution even at slightly acidic pH values is well documented [25,26,27], and they could have a chemical structure unsuitable for interacting with MUPA. Comparing the calibration curves in Figure 9 with that in NaNO_3_ 0.1 M in the absence of any competing metal ion (Figure 6), it is seen that no interaction of uranyl with weak sites takes place, indicating that Ca^2+^ and Mg^2+^ effectively compete with uranyl for access to these sites.

The interferences of other common ions present in natural waters at an electrochemical sensor based on the same receptor, i.e., the MUPA monolayer, have been widely investigated in a previous work [17]. These previous results are shortly reported here for convenience: cations such as Cu^2+^, Zn^2+^, Co^2+^, Ni^2+^, and Pb^2+^ are demonstrated to not interfere up to a 20:1 metal ion/uranyl weight ratio, as well as common anions (nitrates, carbonates, chloride, sulfate, phosphates) up to 1000:1 anion: uranyl weight ratio. Due to the well-known ability to complex uranyl [25], hydrogen carbonate was mainly investigated in [17] and found to interfere only at higher than 200 mg L^−^^1^ concentration, with a 15% reduction in the analytical signal of 50 μg L^−^^1^ uranyl. The influence of possible organic ligands on the uranyl response was investigated too [17]; humic substances did not cause any interference even at concentrations up to 10 mg L^−^^1^ (uranyl: 50 μg L^−^^1^), while strong ligands as EDTA interfere at 1 mg L^−^^1^. Usually, only humic substances are expected to be present in natural waters.

### 3.6. Response to Uranyl in Tap Water

**Figure 10:**
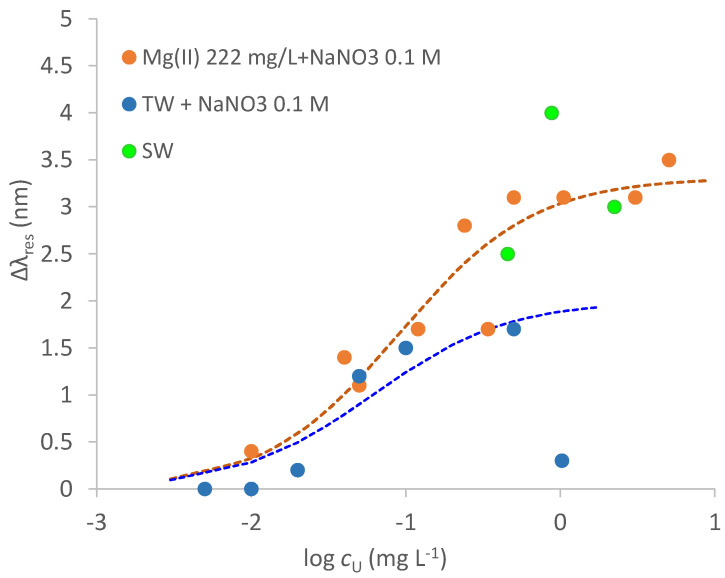
Calibration curves of uranyl in tap water sample (TW) added with NaNO_3_ 0.1 M (blue points); in NaNO_3_ 0.1 M + Mg^2+^ 222 mg L^−^^1^ (orange points) and seawater (SW, green points). Dotted lines are the calculated curves applying Equation (1): orange curve is fitted to orange points; blue curve is fitted to blue points.

As an example of low salinity water, a tap water sample (TW) obtained from the sink of the Analytical Chemistry laboratory in Pavia was considered. The TW composition is: pH = 7.9, 42 mg L^−^^1^ calcium, 8.3 mg L^−^^1^ magnesium, 12 mg L^−^^1^ sodium, 84 mg L^−^^1^ carbonate and a negligible concentration of uranium, <0.1 μg L^−^^1^ (by ICP-MS measurements). To buffer the ionic strength of the considered samples, the TW sample was added with 0.1 M NaNO_3_. Figure 10 reports the calibration curve of uranyl in this media, compared with the standardization curve obtained in synthetic samples containing Mg^2+^ at a much higher concentration than in the TW sample.

The curve of the TW sample and that of the synthetic solution of NaNO_3_ 0.1 M containing Mg^2+^ 222 mg L^−^^1^ appear to be similar up to 0.1 mg L^−^^1^. Actually, the fitting parameters for the TW sample (fitting of the points up to 0.1 mg L^−^^1^) reported in Table 3 (last line) agree pretty well with those of synthetic solutions of Mg^2+^. However, for the TW sample, an unpredictable behavior happens at uranyl concentration higher than 0.1 mg L^−^^1^ since the Δ*λ* value collapses to a very low value. This behavior can be ascribed to the very complex uranyl speciation at slightly basic pH, particularly in the presence of calcium and carbonates [27]. However, such high uranyl concentrations are not usually met in environmental waters, so that the proposed sensor can be successfully applied to the uranyl analysis at common concentrations in low salinity waters.

As a first trial, three points of a dose–response curve for a sea water sample, obtained from the Tyrrhenian sea near Naples, not added with NaNO_3_ 0.1 M, are reported in Figure 10 (green points). The most remarkable difference with the tap water examined above is that a greater red shift of *λ*_res_, most probably ascribable to the much higher RI of seawater (*n* = 1.34), due to the very high concentrations of different salts, in particular sodium chloride, at a salinity of 35 g L^−^^1^ and 20 °C [28]. Moreover, increasing uranyl concentrations produce a blue shift of the resonance wavelength. Further investigation is required, particularly a thorough assessment of the seawater sample composition, to fully understand these observations.

## 4. Conclusions

The D-shaped optical platform on a multimode plastic optical fiber proposed some years ago by our research group is demonstrated to be suitable as an optical sensing tool for uranyl detection in waters of environmental interest at the concentrations usually present in these samples. The considered MRE, MUPA, is a molecule able to bind the uranyl ions strongly and, at the same time, can be steadily linked to the gold surface of the platform by a simple procedure. The selection of this molecular recognition element is crucial for reaching low detection limits and high selectivity. The selectivity of the platform for uranyl with respect to other metal ions widely present in environmental waters is good so that a sufficiently low detection limit for the direct detection of uranyl in environmental waters can be achieved with only a very mild treatment of the sample, such as the addition of an ionic strength buffer.

## Figures and Tables

**Table 1:** Parameters of the dose–response curve fitted to Equation (2), taking into account the existence of strong sites (S) and weak sites (W).

Site	Δ*λ*_max_ /nm	SE Δ*λ*_max_ /nm	*K*_d_ /mg L^−1^	SE *K*_d_ /mg L^−1^	*K*_aff_ /M^−1^	Sens. /nm mg^−1^ L	LOD /mg L^−1^	Adj. R^2^	HL /mg L^−1^
S	2.11	0.37	0.013	0.004	1.84·10^7^	162.8	0.007	0.747	0.011
W	7.66	0.31	4.69	0.32	5.08·10^4^	1.6	0.44	0.985	4.6

SE: standard error; HL: higher quantification limit, calculated as (Δ*λ*_max_-SE)/Sens.

**Table 2:** Parameters of the calibration curves of Ca^2+^ and Mg^2+^ at SPR-POF/MUPA in 0.1 M NaNO_3_ aqueous solution.

Cation	Range	Δ*λ*_max_ /nm	SE Δ*λ*_max_ /nm	*K*_d_ /mg L^−1^	*K*_aff_ /M^−1^	Sens. /nm mg^−1^ L	LOD /mg L^−1^	Adj. R^2^
Ca(II) (red shift)	saturation at 300 mg L^−1^	2.99	0.53	0.09	1·10^3^	0.075	16.6	0.86
Mg(II) (blue shift)	saturation at 10 mg L^−1^	3.93	0.29	0.39	1·10^5^	9.95	0.085	0.96

**Table 3:** Parameters of the calibration curves of uranyl at SPR-POF/MUPA in 0.1 M NaNO_3_ aqueous solution in the presence of Ca^2+^ and Mg^2+^ reported in Figure 9, and in tap water (TW) added with 0.1 M NaNO_3_ reported in Figure 10.

Conditions	Δ*λ*_max_ /nm	SE Δ*λ*_max_ /nm	*K*_d_ /mg L^−1^	*K*_aff_ /M^−1^	Sens. /nm mg^−1^ L	LOD /mg L^−1^	Adj. R^2^
NaNO_3_ 0.1 M	3.79	0.49	0.01	2.93·10^7^	466.5	0.0025	0.72
NaNO_3_ 0.1 M + Mg^2+^ 222 mg L^−1^ pH = 2	3.31	0.14	0.09	2.61·10^6^	36.3	0.007	0.87
NaNO_3_ 0.1 M + Ca^2+^ 625 mg L^−1^ pH = 6	2.99	0.42	0.025	1.12·10^7^	118	0.007	0.66
TW + NaNO_3_ 0.1 M	2.07	0.29	0.061	3.77·10^6^	33.9	0.008	0.94

## Data Availability

Not applicable.
